# Clinicopathological Characteristics of Laterally Spreading Colorectal Tumor

**DOI:** 10.1371/journal.pone.0094552

**Published:** 2014-04-21

**Authors:** Xinhua Zhao, Qiang Zhan, Li Xiang, Yadong Wang, Xianfei Wang, Aimin Li, Side Liu

**Affiliations:** 1 Guangdong Provincial Key Laboratory of Gastroenterology, Department of Gastroenterology, Nanfang Hospital, Southern Medical University, Guangzhou, China; 2 Department of Gastroenterology, Mianyang Central Hospital, Mianyang, China; 3 Department of Gastroenterology, Wuxi City People's Hospital Affiliated with Nanjing Medical University, Wuxi City, China; National Cancer Center, Japan

## Abstract

**Background and Aims:**

Laterally spreading tumor (LST) is a colorectal pre-cancerous lesion. Previous studies have demonstrated distinct LST clinicopathological characteristics in different populations. This study evaluated clinicopathological characteristics of LST in a Chinese population.

**Methods:**

A total of 259 Chinese LST patients with 289 lesions were recruited for endoscopic and clinicopathological analyses.

**Results:**

Among these 289 lesions, 185 were granular type (LST-G), whereas 104 were non-granular type (LST-NG). LST-G lesions were further classified into homogeneous G-type and nodular mixed G-type, while LST-NG lesions were further classified into flat elevated NG-type and pseudo-depressed NG-type. Clinically, these four LST subtypes showed distinct clinicopathological characteristics, e.g., lesion size, location, or histopathological features (high-grade intraepithelial neoplasia and submucosal carcinoma). The nodular mixed G-type showed larger tumor size and higher incidence of high-grade intraepithelial neoplasia compared to the other three subtypes, while pseudo-depressed NG-type lesions showed the highest incidence of submucosal carcinoma. Noticeably, no diffidence was detected between the lesions of homogeneous G-type and flat elevated NG-type with regard to the histopathological features. Histology of the malignancy potential was associated with nodular mixed G-type [OR = 2.41, 95% CI (1.09–5.29); P = 0.029], flat elevated NG-type [OR = 3.49, 95% CI (1.41–8.22); P = 0.007], Diameter ≥30 mm [OR = 2.56, 95% CI (1.20–5.20); P = 0.009], Villous adenoma [OR = 2.76, 95% CI (1.01–7.58); P = 0.048] and serrated adenoma [OR = 6.99, 95% CI (1.81–26.98); P = 0.005].

**Conclusion:**

Chinese LSTs can be divided into four different subtypes, which show distinct clinicopathological characteristics. Morphology, size and pathological characteristics are all independent predictors of advanced histology.

## Introduction

Colorectal cancer is one of most common neoplasms in the world, i.e., it is the second most common cancer diagnosed in women and the third most common in men, accounting for one fourth of worldwide cancer deaths in 2008 [1], [2]. Although precise etiology remains to be defined, an unhealthy life style has been shown to contribute to colorectal carcinogenesis, such as a diet high in fat and red meat, or low in fiber, fruits and vegetables, and is associated with physical inactivity. Due to the rapid adoption of Westernized lifestyles, there is a rapid increase in colorectal cancer incidence in Asia-Pacific regions, including China [3]. To date, endoscopy is frequently used for the early detection of colorectal cancer to effectively prevent late stage cancers. However, in recent years, nonpolypoid colorectal cancer has drawn much attention [4]–[8]. Laterally spreading tumor (LST) of the colorectum is a large and relatively flat neoplastic lesion, which typically extends laterally rather than vertically along the colonic wall [9]–[11] and belongs to the class nonpolypoid colorectal neoplasia. LST is defined as a lesion greater than 10 mm in diameter [12], [13]. Recent studies have indicated that LSTs represents 17.2% of advanced colorectal neoplasia [14] and that LST lesions may develop high-grade intraepithelial neoplasia (HIGN) with an incidence rate ranging from 20.9% to 33.8% [15], [16]. LSTs can also develop into a deeper submucosal invasive cancer [15]–[18]. Thus, the use of colonoscopy for LST diagnosis is crucial to effectively prevent colorectal cancer. LSTs can be treated with endoscopic resection (such as endoscopic mucosal resection, piecemeal endoscopic mucosal resection, or endoscopic submucosal dissection) or surgery to eliminate the late stage of colorectal cancer [10].

However, diagnosis and subtypes of LSTs are complicated because LSTs grow along the surface of the intestine with a low vertical axis that extends laterally along the luminal wall. According to their morphological features, LSTs can be classified into 2 types and 4 subtypes, i.e., granular type (LST-G), including homogeneous G-type and nodular mixed G-type; non-granular type (LST-NG), including flat elevated NG-type and a pseudo-depressed NG-type [5], [7]. Previous studies have suggested distinct genetic characteristics exist between LST-G and LST-NG types [19], [20] and that there are distinct incidences for the development of submucosal invasive carcinoma among these four subtypes [15], [17], [21]. Furthermore, previous studies have shown distinct clinicopathological LST characteristics (for example, morphology, colorectal localization, histopathological feature, and incidence of submucosal invasion) in different populations [14], [16], [22]. In this study, we retrospectively collected LST case histories and investigated their clinicopathological characteristics in a Chinese population.

## Materials and Methods

### Study population

The study population included 38,050 (male: female was 21291:16759) consecutive patients who underwent complete colonoscopy in The Endoscopy Center of Nanfang Hospital between January 2001 and August 2011. The inclusion criteria were as follows; i) age was over 20 years old, and ii) patients did not have inflammatory bowel disease, familial adenomatous polyposis, or serrated polyposis.

**Figure 1:**
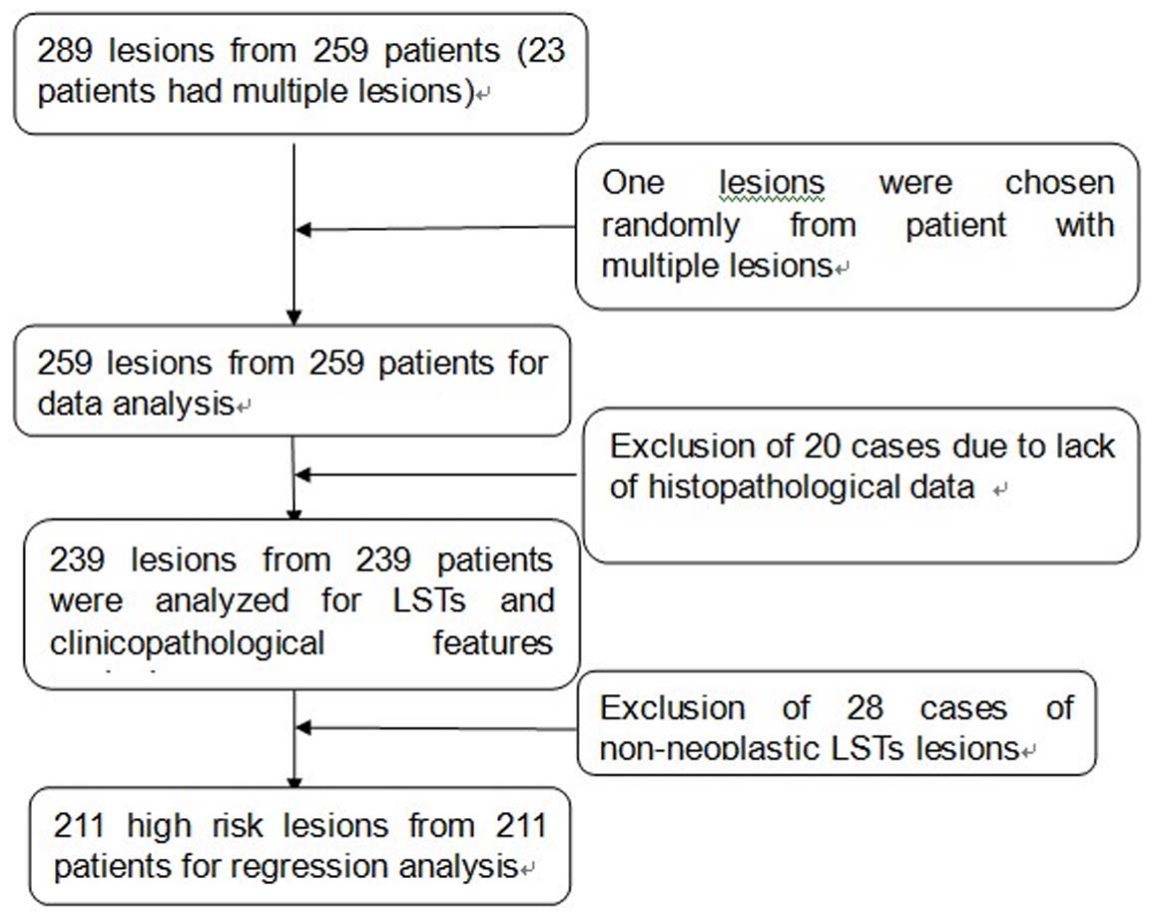
Study design (patient flow chart of the study).

Among this cohort of patients, a total of 259 LST patients were found. Of these 259 patients, 18 had two lesions, 3 had three lesions, and 2 patients had four lesions. The written informed consents were obtained from all the patients before endoscopic procedure. The protocol for this study was approved by the Ethics Committee of Nanfang Hospital (Permission No.: 2011–094). For those patients with multiple lesions, we randomly selected one lesion per multiple lesion patient. Moreover, 20 cases were excluded due to lack of histopathological data; thus, a total of 239 lesions from 239 patients were included for statistical analysis. A further binary logistic regression analysis was performed for 211 of the 239 cases of these high-risk lesions (Fig. 1) with 28 cases excluded as they were non-neoplastic lesions (hyperplastic polyps),

Clinicopathological data were retrospectively retrieved from patients' medical records, including the patient's age, gender, colonoscopy graphic reports, and histopathology. We used the Dizhongtian computerized patient record system (Shenzhen Dizhongtian Electronic Technique Co., Ltd.) to review data on all patients with LSTs. This system is commercial software to help us to maintain all patients' data.

### Evaluation of endoscopic data

All patients received a complete colonoscopy by using one of the electronic video-colonoscope, i.e., CF-230I, CF-240AI, CF-240I, CF-Q240ZI, CF-H260AI, and CF-H260AZI (Olympus, Japan). Experienced endoscopists, who had conducted more than1,000 cases per year during the past 5 years, made the diagnosis and classification of the LSTs. Specifically, when a lesion was detected, 0.4% indigo carmine was used to stain the tissue and then subjected to colonoscopic observation. During the colonoscopy, LSTs are viewed as flat lesions with a diameter greater than 10 mm. Classification of LST subtypes was based on colonoscopic observations according to the Paris classification [12], i.e., homogeneous G-type is 0-IIa lesion; nodular mixed G-type can be a mixture of 0-IIa and 0-Is lesions; flat elevated NG-type is 0-IIa lesion; pseudo-depressed NG-type can be mixture of 0-IIa and 0-IIc lesions [12]. Kudo et al divides pit pattern into six groups: types I, II, III-s, III-L, IV and V [23].

### Histopathological evaluation of LST lesions

LST lesions were histopathologically evaluated by a digestive pathologist based on the 2010 WHO-developed definition of colorectal serrated lesions and updated Vienna classification of gastrointestinal epithelial tumors. Colorectal serrated lesions were classified into hyperplastic polyps, sessile serrated adenoma/polyp with or without cell dysplasia, and traditional serrated adenoma. Intraepithelial neoplasia was classified as a low-grade vs. high-grade lesion. Lesions with “intramucosal” or “in situ” carcinoma were included in high-grade intraepithelial neoplasia (HGIN). Serrated adenoma with any conventional cytological dysplasia was considered as an “advanced” polyp with clinical significance similar to high-grade dysplasia in conventional adenoma [24]. Lesions with tumor cells found in sub-mucosa or deeper tissues were defined as invasive carcinoma. High-grade intraepithelial neoplasia and submucosal carcinoma were defined as advanced histology.

### Treatment of patients

Patients underwent an endoscopic mucosal resection (EMR) [including endoscopic piecemeal mucosal resection (EPMR)] or endoscopic submucosal dissection (ESD) or surgical colectomy. Surgical treatment was recommended for lesions with an invasive pit pattern and no signs of lifting after submucosal injection, or for lesions whose size/location was not suitable for endoscopic therapy. Additional surgery was recommended for patients who had extensive invasive lesions after the endoscopic procedure or relapsed after other treatment.

### Statistical analyses

Quantitative data were expressed as mean ± standard deviation and analyzed using the Student's t-test or one-way analysis of variance. Qualitative data were expressed using ratios and analyzed by the Pearson chi-square test or Fisher's exact test. Kruskal-Wallis H-test was used for nonparametric data. Binary logistic regression was used to analyze risk factors of development of LSTs to advanced histology lesions. All data were analyzed using SPSS 13.0 for Windows (SPSS, Chicago, IL, USA) and a *p* value<0.05 was considered statistically significant.

## Results

### Clinical characteristics of patients

The median age of the 259 patients was 62 years old (ranging between 26 and 93 years old). There was no age difference between male and female patients (t = 0.60, *p* = 0.549; Table 1). Endoscopic evaluation revealed that 185 lesions (64.01%) were G-type, including 75 homogeneous G-type (25.95%) and 110 nodular mixed G-type (38.06%) lesions; 104 lesions were NG-type (35.99%), including 94 flat elevated NG-type (32.53%) and 10 pseudo-depressed NG-type (3.46%) lesions. Of those, 103 lesions (35.6%) were localized in the proximal colon, including 18 in the cecum, 47 in the ascending colon, and 38 in the transverse colon. A further 186 lesions were localized in the distal colon (64.4%), including 13 in the descending colon, 43 in the sigmoid colon, and 130 in the rectum. The average size of the LST lesions at the proximal colon (21.82±10.82 mm) was significantly smaller than in the distal colon (35.90±22.38 mm; t = 7.106, *p*<0.0001; Table 1). Moreover, 118 patients (45.9%) showed other colon lesions, including non-neoplastic polyp lesions, adenoma, or colorectal cancer (Table 1).

**Table 1:** Demographic data of patients with laterally spreading tumors (LST) and endoscopic characteristics and treatment regimes.

Clinical-pathological characteristics	Subcategory	n (%)
Sex	Males: female	145:114
Age, yrs.	Median age, (range)	62 (26 to 93)
	Male	61 (28 to 90)
	Female	61 (26 to 93)
LST type and subtype		
	Granular-LSTs	185 (64.0)
	LST-G-H	75 (26.0)
	LST-G-MX	110 (38.1)
	Nongranular-LSTs	104 (36.0)
	LST-NG-F	94 (32.5)
	LST-NG-PD	10 (3.5)
Size, mm, mean ± SD	Average tumor size	30.88±20.22
	Proximal colon	21.82±10.82
	Distal colon	35.90±22.38
Tumor location		
	Proximal colon	103 (35.6)
	Cecum	18 (6.2)
	Ascending colon	47 (16.3)
	Transverse colon	38 (13.1)
	Distal colon	186 (64.4)
	Descending colon	13 (4.5)
	Sigmoid colon	43 (14.9)
	Rectum	130 (45.0)
Concomitant		118 (45.6)
	Hyperplastic polyp	31 (12.0)
	Adenoma	58 (22.4)
	Multiple adenoma	22 (8.5)
	Colorectal cancer	3 (1.2)
	Adenoma and cancer	4 (1.5)
Treatment		
	No treatment	20 (6.9)
	APC	2 (0.7)
	EMR(EPMR)	207 (71.6)
	ESD	22 (7.6)
	Colectomy	29 (10.0)
	Additional surgery	9 (3.1)

SD, standard deviation; LST-G-H, homogeneous G-type; LST-G-MX, nodular mixed G-type; LST-NG-F, flat elevated NG-type; LST-NG-PD, pseudo-depressed NG-type; APC, argon plasma coagulation; EMR endoscopic mucosal resection; EPMR, endoscopic piecemeal mucosal resection. ESD, endoscopic submucosal dissection.

Following the initial evaluation, 183 patients (with a total of 207 lesions) received EMR (including EPMR), and the remaining lesions were further treated with Argon plasma coagulation (APC). Nineteen patients (22 lesions) received ESD treatment with one case of delayed perforation and one small perforation, which was then successively addressed by conservative treatment. Surgical resection of lesions was used for 28 patients (29 lesions), i.e., five patients (6 lesions) with a tumor size greater than 90 mm; two lesions with 35 mm diameter but not suitable for endoscopic treatment because of their locations; and eleven patients with Vn or Vi pit patterns, which indicated submucosal invasive carcinoma which was confirmed by post-operative pathological observation; two patients with submucosal (SM2) lesions confirmed using an ultrasound endoscopic test prior to the surgery; four patients with complicated cancer at other locations of the colon. Three patients who were suitable for endoscopic treatment initially asked for surgical treatment, as did one patient with local recurrence after EPMR treatment. Seven patients (8 lesions) received additional surgery due to submucosal invasive carcinoma after endoscopic based treatment (5 cases of EPMR and 3 cases of ESD). Two patients received APC treatment due to adhesive lesions with negative signs of lifting, which did not fit the indications of endoscopic treatment (Table 1).

However, 19 patients (20 lesions) abandoned treatment due to contraindications or for unknown reasons. One patient had decompensated liver cirrhosis, one had poor cardiopulmonary function and could not accept endoscopic or surgical treatment, another (2 lesions) had late stage rectal cancer and abandoned the treatment, and the remaining 16 patients abandoned their treatment for unknown reasons.

### Histopathological diagnosis of patients

Histopathological evaluation revealed 239 lesions of LSTs, including 43 (18.0%) cases of tubular adenoma, 94 (39.3%) cases of tubular villous adenoma, 57 (23.8%) cases of villous adenomas, 17 (7.1%) cases of serrated adenomas, and 28 (11.7%) cases of hyperplastic polyps. Of these 239 lesions, 101 (42.3%) lesions were LGIN, 86 (36.0%) lesions were HGIN, and 24 (10.0%) lesions were submucosal invasive carcinoma. The size of homogeneous G-type LSTs and flat elevated NG-type lesions with advanced histology were markedly larger than those of low-grade and non-cancerous lesions. However, this was not observed in the nodular mixed G-type lesions. Moreover, if the size of flat elevated NG-type lesions was greater than 20 mm and the size of homogeneous G-type was greater than 30 mm, the risk of advanced histology was markedly increased (Table 2). The median age of patients with advanced histology was 61 years of age (ranging between 26 and 90 years old), which was similar to the age of patients with Non-advanced histology (60 years old, ranging from 26 to 86 years old).

**Table 2:** Advanced histology and LSTs size.

LSTs Subtype	Histology	n(239)	Diameter,mm, mean ± SD	10–19 mm,n	20–29 mm,n	30–39 mm,n	40–49 mm,n	50–59 mm,n	≥60 mm,n
LST-G-H	Advanced	17	48.3±26.1	4	1	1	1	3	7
	Nonadvaced	45	25.4±12.5	16	15	7	2	4	1
LST-G-MX	Advanced	60	45.9±22.9	3	11	14	7	5	20
	Nonadvaced	39	38.5±23.4	1	11	13	6	3	5
LST-NG-F	Advanced	28	23.9±11.0	11	8	8	0	0	1
	Nonadvaced	43	16.6±6.3	28	13	2	0	0	0
LST-NG-PD	Advanced	5	18.4±5.0	2	3	0	0	0	0
	Nonadvaced	2	17.5±3.5	1	1	0	0	0	0

In 24 patients with submucosal invasive carcinoma, the average size of the lesions was 39.79±25.10 mm and the major subtype was the nodular mixed G-type (14 cases, 58.3%). Nineteen (79.2%) lesions were localized at the distal colon. Eighteen cases (75%) were diagnosed as early stage colorectal cancer (including T1N0M0 and T2N0M0). Thus, all of these 24 patients received surgery (Table 3).

**Table 3:** Clinicopathological characteristics of LSTs with submucosal invasive.

Clinicopathological characteristics	Subcategory	Case
Sex	male: female	10:14
Age, yrs.	median age (range)	64.5 (40–79)
LSTs subtype		24
	LST-G-H	2
	LST-G-MX	14
	LST-NG-F	4
	LST-NG-PD	4
Size	mean ± SD (mm)	39.79±5.12
Tumor location		
	Proximal colon	5
	Distal colon	19
TNM		
	T1N0M0	12
	T2N0M0	6
	T3N0M0	1
	T3N1M0	1
	T3N0M1	1
	T4N1M0	2
	T4N1M1	1

TNM, Tumor, Node, and Metastasis staging followed NCCN (National Comprehensives Cancer Network) clinical practice guidelines in oncology, colon cancer 2012.

**Figure 2:**
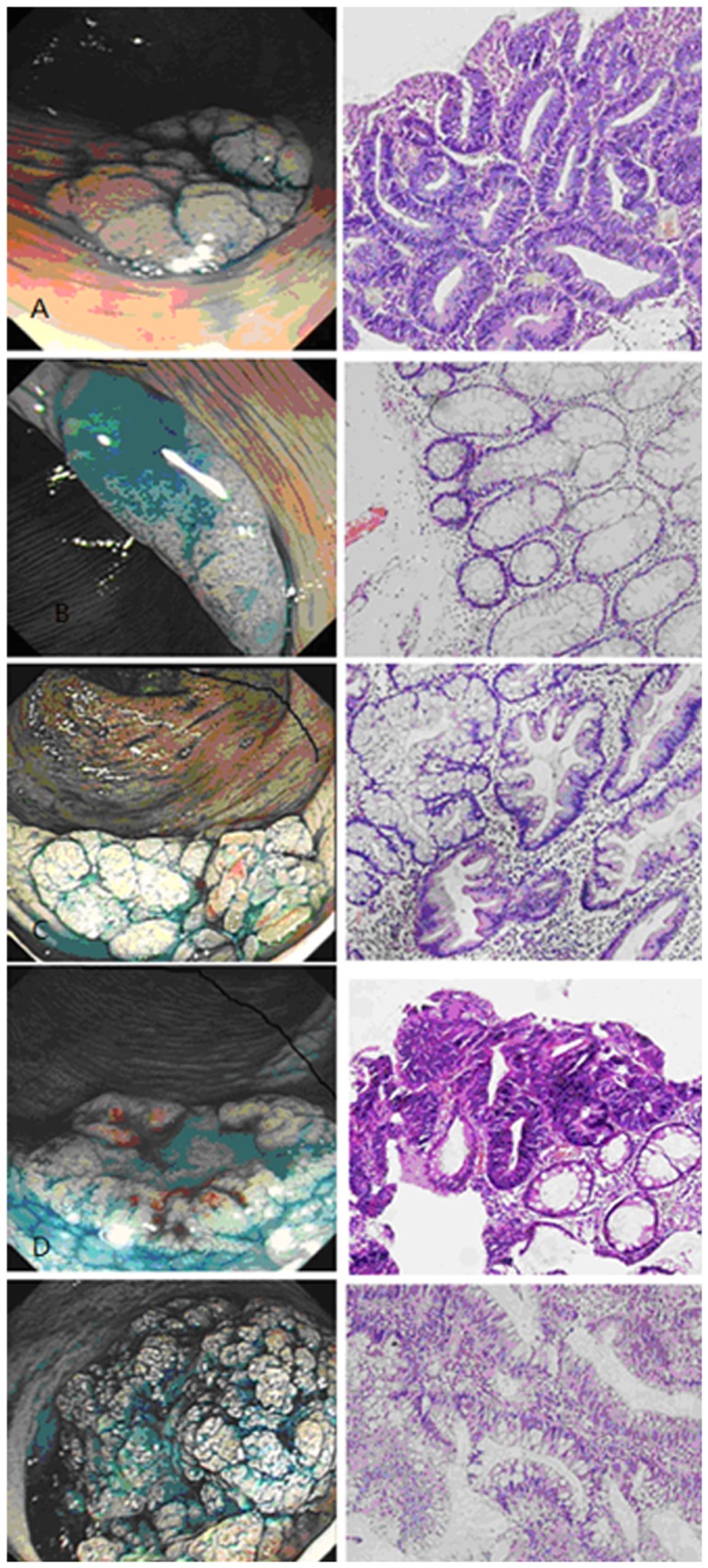
Endoscopic LST imaging and H&E staining of LST tissue specimens. A, Homogeneous G-type tubular adenoma; B, Flat elevated NG-type hyperplastic polyps; C, Homogeneous G-type serrated adenoma; D, Nodular mixed type tubular villous adenoma with malignancy. E, Pseudo-depressed NG-type villous adenoma with malignancy; Resected tissue specimens were retrieved and immediately fixed in a 10% buffered formalin solution, and subjected to hematoxylin and eosin staining; 200× magnification.

Furthermore, lesions of nodular mixed G-type had an average size of 43.0±22.1 mm, which was much larger than that of the other three subtypes (*p*<0.0001). However, there was no statistically significant difference between size of flat elevated NG-type and pseudo-depressed NG-type lesions (*p* = 0.973). Moreover, 53.2% (33/62) of the homogeneous G-type lesions and 38 (53.5%) of the flat elevated NG-type lesions were localized at the proximal colon. In addition, 83 (83.8%) nodular mixed G-type lesions and 6 (85.7%) pseudo-depressed NG-type lesions were localized at the distal colon (Table 4). Histopathological evaluation of these lesions revealed that there was no diffidence in histology between homogeneous G-type and flat elevated NG-type lesions (*p*>0.05). However, these two subtypes showed a higher incidence in the development of tubular adenoma and hyperplastic polyps compared to the nodular mixed G-type and pseudo-depressed NG-type lesions (*p*<0.05). However, components of villous adenoma were associated with nodular mixed G-type rather than the other subtype LSTs. Serrated adenomas were identified in five (8.1%) homogeneous G-type LSTs, four (4.0%) nodule-mixed G-type LSTs, and eight (11.3%) flat elevated NG-type LSTs. Pseudo-depressed NG-type tumors did not contain serrated adenoma components, although the overall distribution of serrated adenoma did not differ significantly among the subtypes of LST (*p* = 0.275; Table 4). Lesions of pseudo-depressed NG-type had the highest incidence of submucosal invasion (4/7, 57.1%) among these four subtypes (Table 4), while lesions of nodular mixed G-type had the highest incidence of developing HGIN (46/99, 46.5%) among these four subtypes (Table 4). Fig. 2 shows the endoscopic and histopathological findings in our study according to LST subtype.

**Table 4:** Clinicopathological characteristics of these 239 LSTs.

LSTs subtype	n(239)	Diameter,mm,mean±SD	Location,P-coln∶D-colon	TA	TVA	VA	SA	HPs	HGIN	SM
LST-G-H	62	31.7±20.0	33∶29	16	23	9	5	9	15	2
LST-G-MX	99	43.0±22.1	16∶83	8	50	35	4	12	46	14
LST-NG-F	71	19.5±9.1	38∶33	19	19	8	8	17	24	4
LST-NG-PD	7	18.1±4.4	1∶6	0	2	5	0	0	1	4
P	NA	8.902E-14^a^	8.278E-7^d^	0.002^e^	0.016^e^	1.470E-5^d^	0.275^e^	1.290E-4^e^	0.014^e^	2.956E-5^e^
_Δ_P	NA	2.860E-5^b^	0.973^d^	0.901^d^	0.275^d^	0.576^d^	0.535^d^	0.251^d^	0.225^d^	0.685^e^

TA, tubular adenoma; TVA, tubular villous adenoma; VA, villous adenoma; SA, serrated adenoma; HPs, hyperplastic polyps; HIGN, high-grade intraepithelial neoplasia; SM, submucosal carcinoma; P-colon, proximal colon; D-colon, distal colon.P, compared among the four LST subtypes; _Δ_P, compared between LST-G-H and LST-NG-F.

a , one-way analysis of variance;

b , Student's t-test;

d , Pearson chi-square test;

e , Fisher's exact test.

In addition, we found that 66.7% of nodular mixed G-type lesions were IV pit pattern and 57.1% of pseudo-depressed lesions were V pit pattern (*p*<0.05; Table 5). There was no difference found between homogeneous G-type and flat elevated NG-type lesions with regard to the pit pattern. A total of 51.6% of homogeneous G-type and 47.9% of flat elevated NG-type lesions were III-L pit pattern, which occurred more frequently than the nodular mixed G-type and pseudo-depressed NG-type lesions (*p*<0.001; Table 5).

**Table 5:** Pit patterns and LST subtypes.

LSTs subtype	n (239)	Pit pattern, number of cases (%)					
		I	II	IIIs	III-L	IV	V
LST-G-H	62	1 (1.6)	6 (9.7)	6 (9.7)	32 (51.6)	16 (25.8)	1 (1.6)
LST-G-MX	99	0 (0.0)	5 (5.1)	2 (2.0)	23 (23.2)	66 (66.7)	3 (3.0)
LST-NG-F	71	4 (5.6)	13 (18.3)	2 (2.8)	34 (47.9)	16 (22.2)	2 (2.8)
LST-NG-PD	7	0 (0.0)	0 (0.0)	0 (0.0)	1 (14.3)	2 (28.6)	4 (57.1)
P		NA	0.009^e^	0.056^e^	3.255E-4^e^	2.680E-9^e^	0.004^e^
_Δ_P		0.371^e^	0.156^d^	0.145^e^	0.668^d^	0.660^d^	1.00^e^

P, compared among the four LST subtypes; _Δ_P, compared between LST-G-H and LST-NG-F;

d , Pearson chi-square test;

e , Fisher's exact test.

### Logistic regression analysis

A binary logistic regression analysis revealed that lesion subtype, size, and histopathological feature were all independent predictors of advanced histology (Table 6). Specifically, nodule mixed G-type vs. homogeneous G-type had an OR of 2.41 (95% CI of 1.09–5.29, *p* = 0.029); flat elevated type vs. homogeneous G-type had an OR of 3.49 (95% CI of 1.41–8.22, *p* = 0.007). Lesions with diameters ≥30 mm vs. <30 mm had an OR of 1.26 (95% CI of 1.26–5.20, *p* = 0.009). Villous adenoma vs. tubular adenoma had an OR of 2.76 (95% CI of 1.01–7.58, *p* = 0.048). Serrated adenoma vs. tubular adenoma had an OR of 6.99 (95% CI 1.81–26.98, *p* = 0.005). However, age, gender and tumor location were irrelevant.

**Table 6:** Multivariate analysis of LSTs with advanced histology.

	Advanced histology cases/total cases	OR [95% CI]	p value
LST-G-MX vs. LST-G-H	60/97 vs. 17/53	2.41 [1.09–5.29]	0.029
LST-NG-F vs. LST-G-H	28/54 vs. 17/53	3.49 [1.41–8.22]	0.007
LST-NG-PD vs. LST-G-H	5/7 vs. 17/53	4.91 [0.75–32.17]	0.097
D≥30 mm vs. D<30 mm	67/106 vs. 43/105	2.56 [1.26–5.20]	0.009
D-colon vs. P-colon	88/148 vs. 22/63	0.50 [0.24–1.04]	0.065
TVA vs. TA	47/94 vs. 12/43	1.55 [0.64–3.77]	0.330
VA vs. TA	39/57 vs. 12/43	2.76 [1.01–7.58]	0.048
SA vs. TA	12/17 vs. 12/43	6.99 [1.81–26.98]	0.005

D, diameter; TA, tubular adenoma; TVA, tubular villous adenoma; VA, villous adenoma; SA, serrated adenoma; P-colon, proximal colon; D-colon, distal colon.

## Discussion

LSTs are superficial non-polypoid colorectal tumors with a diameter greater than 10 mm, and are considered pre-cancerous colorectal lesions [8]. In the current study, we demonstrated a similar demographic profile of Chinese LST patients to that of a previous study involving an Italian population with regard to age and gender, although our study showed a slightly higher incidence in male patients and patients older than 60 years of age [15]. However, our data showed that most lesions were localized at the distal colon (64.36%), which differs from previous studies showing that the proximal colon is the most frequent location of LSTs (55.7–80%) [25]–[28]. This is consistent with the result of another study from China, wherein 75.2% of LSTs were located close to the distal colon [29]. This finding further confirmed that different populations have distinct clinicopathological features of LSTs, and may further indicate a characteristic of the Chinese LST population. Further study is required to verify this finding, because variability of endoscopic observation in previous studies showed a false negative rate of 12–17% for adenoma with lesions ≥10 mm [30]. Furthermore, in our current study, we found that 120 patients (46.3%) were concomitant of polypoid colorectal lesions, including 79 patients (30.5%) with cancerous polypoid lesions and 7 patients (2.7%) with colorectal cancer at other locations, indicating that LSTs often occur with concomitants of malignancy. In addition, our current data showed that the incidence of these four subtypes was consistent with previous studies, e.g., 60.8–83% of G-type lesions [15], [16]. However, another study showed that the NG-type is the major subtype (54.36%) based on a study of 1,363 cases, and flat elevated NG-type lesions are the most common subtype [31]. Pseudo-depressed NG-type is the rarest subtype with an incidence of 2.7%–4% reported in previous studies [15], [16], [31]. Indeed, discrepancies did occur, which may be due to the individual experiences and skills of the colonoscopic physicians. For instance, lesions of the NG-type have a smooth surface, which may be mis-diagnosed by an endoscopist, particularly in the proximal colon.

Furthermore, our current study also showed that the lesions of these four subtypes had distinct locations, diameters, histopathological features, potential of malignancy and submucosal invasion. For instance, 66.7% of the nodular mixed G-type lesions occurred at the rectum with an average diameter larger than that of the other three subtypes. The occurrence of nodules was associated with villous adenoma and had incidences of 46.5% and 14.1% respectively of developing further to HGIN and submucosal invasive carcinoma, which is consistent with an earlier study [16]. Granular nodular mixed (88.9%) and nongranular pseudodepressed tumors (85.7%) were more commonly localized at the distal colon. In contrast, homogeneous (53.2%) and flat-elevated tumors (53.5%) were commonly found in the proximal colon, and these results are consistent with another Chinese study [29]. Of the homogeneous G-type lesions, 75.71% were low-grade neoplasia and had the lowest incidence of submucosal invasive carcinoma, which is in agreement with previous studies [27], [28], [32]. Overall, incidences of submucosal invasive carcinoma in these four subtypes were consistent with a previous study that showed incidences of 0.9%, 13.3%, 6.1% and 42.1% for homogeneous G-type, nodular mixed type, flat elevated type and pseudo-depressed type, respectively [19]. Noticeably, no difference was detected between the homogeneous G-type and flat elevated NG-type lesions regarding the development of submucosal invasion and histopathological constitution (higher incidences of tubular adenomas and hyperplastic polyps compared to nodular mixed G-type and pseudo-depressed NG-type lesions). However, there were differences in the clinicopathological features between these two G-LSTs subtypes. The same was also true between the two NG-LSTs subtypes. Our current data suggest that further investigation of sub-differentiated LST lesions is warranted, instead of continuous focus on the original type and subtypes. Precision in morphology classification of LSTs may bring more reliable results.

In addition, colorectal serrated lesions include hyperplastic polyps, sessile serrated adenoma, and traditional serrated adenoma [33]. Sessile serrated adenoma and traditional serrated adenoma are precancerous lesions of colorectal cancer, and 20–35% of cases of sporadic colorectal cancer originate from sessile serrated adenoma [34]. In the current study, we found 17 (17/239, 7.11%) lesions of sessile serrated adenoma/polyp, which was consistent with a previous report [16]. However, we did not find lesions of traditional serrated adenoma. Neither did we find any serrated lesions (hyperplastic polyps or sessile serrated adenomas) in pseudo-depressed type nor was there difference in incidence of sessile serrated adenomas among the other three subtypes. Incidence of hyperplastic polyps in the homogeneous G-type and flat elevated NG-type was much higher than the incidence in nodular mixed G-type, which is consistent with a previous report showing endoscopic features of larger hyperplastic polyps and sessile serrated adenoma, which included flat morphology and location in the proximal colon [35]. Moreover, we found that larger lesions had higher incidences of advanced histology, i.e., lesion sizes of 10–29 mm, 30–59 mm and more than 60 mm showed incidences of 33.3%, 51.3%, and 82.4% for advanced histology, respectively, which is consistent with a previous study [21]. This trend was more obvious for homogeneous G-type and flat elevated NG-type lesions. Regarding histopathological characteristics, serrated adenoma had the highest incidence of HIGN, compared to villous adenoma and tubular adenoma (the lowest), which is in agreement with previous studies [36]. However, villous adenoma had the highest incidence of submucosal invasive carcinoma compared to serrated adenoma and tubular adenoma (the lowest). Logistic regression analysis revealed that morphology, size and pathological characteristics were all independent predictors of advanced histology, which differed from another study showing none of the studied parameters (i.e., age, sex, morphology, location, depressed surface, and large nodule) were independent predictors of high-risk malignancy, suggesting unique clinicopathological characteristics in Chinese LST patients [27]. In addition, in the current study, we also found that pit patterns of lesions were associated with histopathological characteristics and degree of cellular atypia. For instance, III-L is a dominant pit pattern in homogeneous G-type and flat elevated NG-type; IV and V pit patterns were most common in nodular mixed G-type and pseudo-depressed NG-type lesions, respectively. Pit pattern had a value to predict submucosal invasive carcinoma [27], e.g., 50% of pseudo-depressed NG-type lesions were V pit pattern, and pseudo-depressed NG-type lesions had a high incidence of submucosal invasive carcinoma.

Previous studies have demonstrated that LSTs generally have a low incidence of submucosal invasion and that most LSTs can be removed endoscopically [37]. However, additional surgery after endoscopic removal of the lesions is routinely required after submucosal invasive carcinoma is diagnosed by pathological observation. Based on our experience, large homogeneous G-type lesions can be treated with EPMR due to its low submucosal invasion rate. However, complete resection of ESD is a better option for pseudo-depressed NG-type lesions, even for relatively smaller lesions (≤30 mm), since they have a high incidence of submucosal invasion.

Our current study does have limitations. For example, our current study did not provide incidence rates of LSTs in this studied population. Moreover, all of the data were collected retrospectively, and we could not obtain data on relapse after EMR and ESD treatment. In addition, we focused on deep submucosal invasive carcinoma, which may have ignored submucosal invasive lesions with ≤1000 µm depth, resulting in an under estimation of the incidence of submucosal invasive lesions in this LST population. Taking these limitations into consideration, our current study does provide useful data on Chinese LSTs patients, and demonstrated that there were similar LST subtypes compared to other populations; these four LST subtypes had distinct clinicopathological characteristics. A future prospective study will investigate different treatment options for these four types of LSTs.
